# Supplementary material for: The interactome of intact mitochondria by cross-linking mass spectrometry provides evidence for coexisting respiratory supercomplexes
Source: Mol Cell Proteomics. 2017 Dec 8;17(2):216–32. doi: 10.1074/mcp.RA117.000470 (PMC5795388; doi:10.1074/mcp.RA117.000470)
Supplement: Supplemental Data [file supp_RA117.000470_133922_0_supp_23978_fzffwf.zip › spectra_annotation/mito_DR_spectra_annotation/42-1-6-1-14-1.pdf]

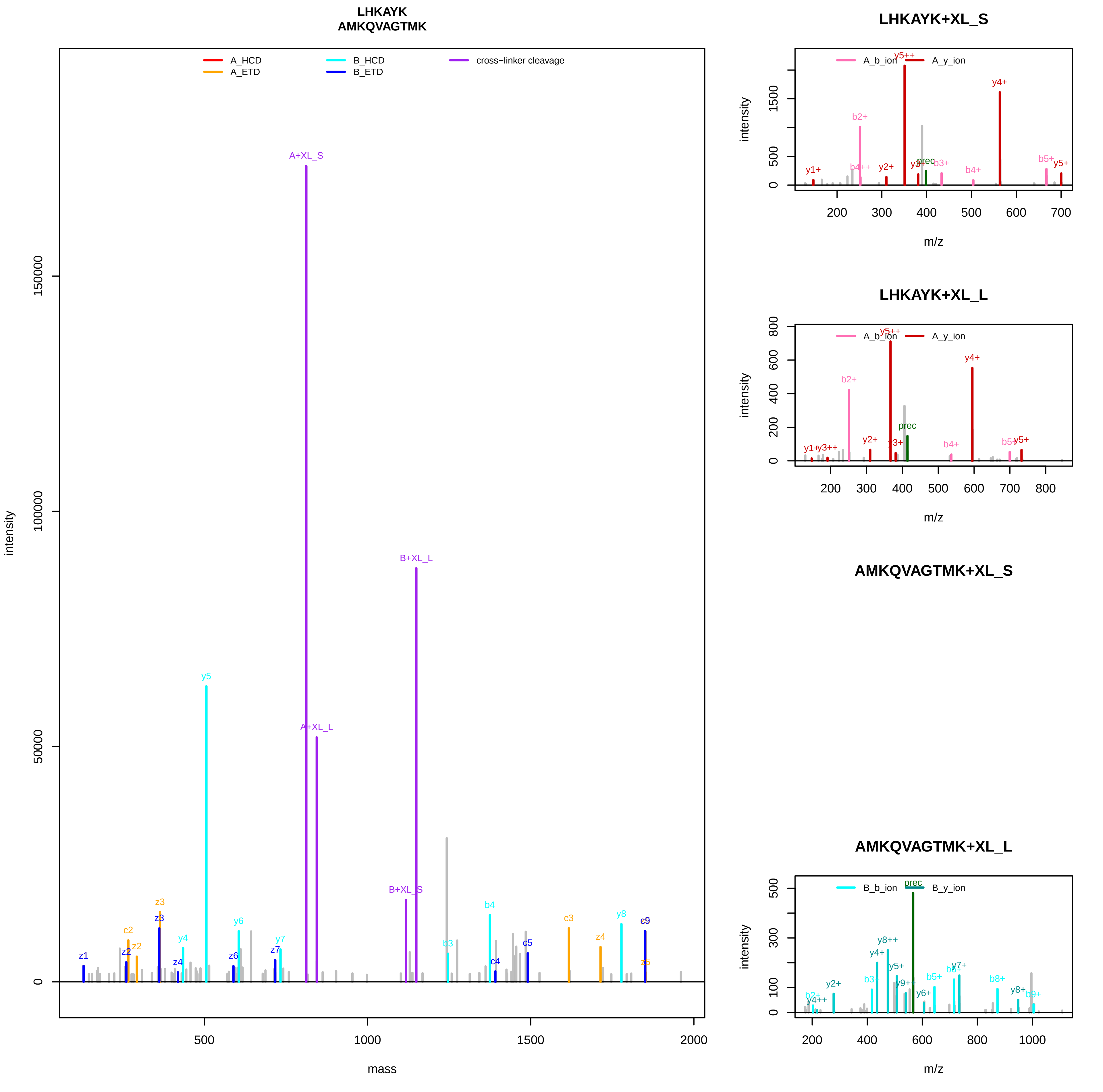

LHKAYK
AMKQVAGTMK
A_HCD
A_ETD
B_HCD
B_ETD
cross−linker cleavage
intensity
mass
0
50000
100000
150000
500
1000
1500
2000
A+XL_S
B+XL_L
A+XL_L
B+XL_S
y5
y6
y7
y8
y4
b3
b4
z1
z2
z3
z4
z6
z7
c2
c3
c4
c5
c9
z5
LHKAYK+XL_S
A_b_ion
A_y_ion
y5++
y4+
b2+
b4++
y2+
y3+
prec
b3+
b4+
b5+
y5+
y1+
m/z
LHKAYK+XL_L
y1+
y3++
AMKQVAGTMK+XL_S
AMKQVAGTMK+XL_L
B_b_ion
B_y_ion
y8++
y4+
y5+
y9++
y6+
b5+
b6+
y7+
b8+
y8+
b9+
b3+
b2+
y4++
y2+
